# A bibliometric analysis on drilling of composite materials using a systematic approach

**DOI:** 10.1016/j.heliyon.2024.e37282

**Published:** 2024-09-02

**Authors:** Pierre Rahme

**Affiliations:** Department of Industrial and Mechanical Engineering, Lebanese American University, Lebanon

**Keywords:** Composite materials, CFRP, Drilling, Delamination, Bibliometric analysis

## Abstract

The increasing use of composite materials across various industries necessitates a detailed understanding of the machining processes involved in their production. This study presents a comprehensive bibliometric analysis focused on the drilling of composite materials, aiming to identify key trends, advancements, and research gaps in this critical area. The novelty of this research lies in its systematic approach to mapping the intellectual landscape surrounding drilling processes, particularly emphasizing underexplored topics such as hybrid composites and alternative drilling techniques like laser and ultrasonic methods. An analysis of 927 relevant publications using the Scopus database and VOSviewer software revealed significant insights into the concentration of research on Carbon Fiber Reinforced Polymer (CFRP) and Glass Fiber Reinforced Polymer (GFRP), while highlighting a lack of focus on sustainable drilling practices and real-time defect detection methods. The results suggest that while optimization of traditional drilling parameters is well-covered, there is a pressing need for future research on tool wear mechanisms and environmentally friendly drilling approaches. These findings provide valuable guidance for improving the efficiency, precision, and sustainability of drilling processes, thereby enhancing the industrial application of composite materials.

## 1 Introduction

Drilling is frequently practiced machining process in modern industry owing to the need for component assembly in composite structures [1]. Composite materials, composed of two or more distinct materials with different physical or chemical properties, offer a unique set of advantages, including high strength-to-weight ratio, corrosion resistance, and enhanced durability [2]. The drilling of composite materials has emerged as a thrilling frontier in engineering and manufacturing, pushing the boundaries of what is possible in industries ranging from aerospace to automotive. However, the drilling of these materials is complicated due to their exceptional anisotropic [3]. Drilling of composite materials is a challenging machining process that requires careful selection of cutting parameters, tool geometry, and material properties [4]. Indeed, drilling of composite materials can cause problems such as delamination, fiber pull-out, thermal damage, hole oversize, and burr formation, which can compromise the structural integrity and performance of the composite parts ([5], [6]). Therefore, many researchers have investigated the effects of different factors on the drilling behavior and outcomes of composite materials ([[7], [8], [9], [10], [11]]). For instance, these studies have focused on traditional drilling techniques for Carbon Fiber Reinforced Polymer (CFRP) and Glass Fiber Reinforced Polymer (GFRP), identifying issues such as delamination, fiber pull-out, thermal damage, hole oversize, and burr formation, which compromise structural integrity and performance. While these studies have significantly contributed to understanding the challenges in drilling composite materials, there is still a need to explore how different material properties and drilling parameters interact to cause these defects. Other researchers have proposed various methods and strategies to improve drilling performance ([[12], [13], [14], [15], [16]]). Also, significant focus has been placed on achieving delamination-free drilling, which is critical for maintaining the structural integrity of composites ([[17], [18], [19], [20], [21], [22], [23]]). However, these strategies often focus on specific cases, and the generalizability of the findings across different composite materials remains underexplored. Moreover, while the optimization of drilling parameters is well-studied, the literature lacks a comprehensive approach to understanding tool wear mechanisms and their real-time detection, an area that is critical for enhancing process reliability and efficiency. On the other hand, other studies include the development of specialized drill bits and techniques to reduce drilling-induced damage ([[24], [25], [26], [27]]). Through these efforts, advancements have been made in enhancing hole quality and extending the application of composite materials in various industries ([7], [10], [13]). More, recent studies have increasingly focused on the use of automation and real-time monitoring systems to optimize drilling processes and monitor tool wear. Barik et al. [28] demonstrated the effectiveness of wavelet packet analysis in predicting the wear of TiAlN- and TiN-coated carbide tools during the drilling of bidirectional CFRP laminates. Their study highlights the potential of using thrust-torque signatures for real-time tool wear prediction and process optimization. Despite these advancements, there is a clear gap in integrating these systems with sustainable drilling practices, such as eco-friendly lubrication and recyclability of drilled composites, which are essential for the broader adoption of these technologies. Recently, sensor-based signal analysis has emerged as a crucial tool for monitoring the drilling process. The effectiveness of wavelet packet analysis in predicting the quality of drilled holes in carbon fiber-reinforced plastics has been demonstrated [29]. By extracting significant features from force-torque signals, these methodologies enable real-time process adjustments, thereby reducing the risk of rework or rejection. Nevertheless, recent advancements in hybrid composite-metal stack drilling have significantly enhanced modern fabrication procedures. A notable study by Xu et al. [30] investigates the effects of different cutting sequence strategies on temperatures, surface morphologies, and tool wear when drilling CFRP/Ti6Al4V stacks. Their findings reveal critical insights into optimizing drilling parameters to improve efficiency and surface quality in hybrid composite-metal stacks. Several authors investigated different factors on drilling hybrid composite-metals stacks (HCMS) such as high-speed cutting, cooled compressed air, and stack order ([31], [32]). To investigate delamination during drilling of CFRP/metal stacks, analytical models are proposed to predict critical thrust force, considering the elastic deformation of both materials [33].

In this paper, a bibliometric analysis, that employs quantitative and qualitative methods to map the intellectual landscape surrounding drilling of composite material, is presented. This study aims to fill the above mentioned gaps by conducting a comprehensive bibliometric analysis of the drilling of composite materials. Key metrics such as citation counts, authorship patterns, and institutional collaborations are examined to identify influential contributors and collaborative networks within the field. By systematically analyzing 927 relevant papers, this study identifies key contributors, thematic trends, and research gaps, focusing on underrepresented areas such as hybrid composites, alternative drilling methods like laser and ultrasonic drilling, and the integration of environmental sustainability into drilling processes. Additionally, keyword co-occurrence and clustering analyses are employed to unveil thematic trends and emerging research directions. Unlike previous studies that primarily address specific aspects of drilling challenges, this analysis provides a holistic view, offering insights into optimizing drilling practices across a broader range of composite materials. Furthermore, the study highlights the need for future research on real-time defect detection, tool wear mechanisms, and sustainable drilling practices, thereby setting the stage for innovative advancements in this field. Through this bibliometric exploration, the paper aims to provide researchers, practitioners, and policymakers with valuable insights into the current state of knowledge in composite material drilling. The findings are expected to contribute to the identification of research gaps, inform future research agendas, and facilitate collaborative efforts in advancing the efficiency, precision, and sustainability of drilling processes applied to composite materials. This study identifies several blank spots in the existing literature. These include the need for more in-depth research on tool wear mechanisms, the development of real-time monitoring systems for defect detection, and the exploration of sustainable drilling practices. Addressing these gaps will be crucial for advancing the precision, efficiency, and environmental sustainability of drilling processes in composite materials. Ultimately, this bibliometric analysis seeks to foster a deeper understanding of the evolving landscape in composite material drilling and promote innovation in materials processing technologies. The study is useful for academics to understand the most influential research, countries, and journals on the topic of composite materials drilling.

## 2 Background of the study

Existing drilling technologies for drilling of composite materials may be grouped into four main categories namely conventional drilling using cutting tools, ultrasonic drilling, Abrasive Water Jet drilling (AWJ), and laser drilling.

• **Conventional drilling**

One of the main factors that influences the drilling of composite materials is the cutting tool [34]. The tool material, geometry, and coating can affect the tool wear, cutting forces, heat generation, chip formation, and hole quality [35]. Different types of tools, such as twist drills, step drills, core drills, spade drills, and special drills, have been used for drilling of composite materials, each with its own advantages and disadvantages [36]. The tool material should have high hardness, wear resistance, and thermal conductivity to withstand the abrasive and heterogeneous nature of the composite materials [37]. The tool geometry, such as point angle, helix angle, rake angle, chisel edge, and margin, should be optimized to reduce the thrust force, torque, and delamination ([38], [39]). The tool coating, such as diamond, can enhance the tool life, reduce the friction and adhesion, and improve the surface finish ([[40], [41], [42], [43]]). Another important factor that affects the drilling of composite materials is the cutting parameters, such as spindle speed, feed rate, and drill diameter [[44], [45], [46]]. The cutting parameters should be selected based on the tool geometry, material properties, and desired hole quality -[[47], [48], [49], [50], [51]]. The spindle speed and feed rate can influence the cutting temperature, cutting forces, tool wear, chip evacuation, and hole accuracy [52]. Generally, high spindle speed and low feed rate can reduce the delamination and improve the surface finish, but they can also increase the tool wear and thermal damage [53]. Larger drill diameters can cause higher cutting forces, torque, and delamination, but they can also reduce the hole oversize and burr formation [54]. The material properties, such as the fiber type, fiber orientation, fiber volume fraction, matrix type, and laminate stacking sequence, can also affect the cutting forces, delamination, and hole quality. There are other factors that can influence the drilling of composite materials, such as the drilling method, the cooling condition, and the tool wear.

• **Ultrasonic drilling**

Ultrasonic drilling has emerged as a promising technique for overcoming delamination challenges by utilizing high-frequency vibrations to facilitate material removal with minimal damage [55]. Several authors have utilized ultrasonic vibration-assisted drilling to reduce defects in composite materials [[56], [57], [58], [59], [60], [61]]. Recent advancements in ultrasonic drilling techniques have focused on improving drilling efficiency, precision, and minimizing damage to composite materials. One approach involves optimizing the frequency, amplitude, and feed rate of ultrasonic vibrations to achieve better control over the drilling process [62], [63]. Additionally, researchers have explored the use of advanced tool geometries, such as helical or serrated edges, to enhance material removal rates and reduce tool wear [64], [65]. Moreover, the integration of real-time monitoring systems, such as acoustic emission sensors and thermal imaging, has enabled better process control and detection of potential defects during drilling [[66], [67], [68], [69], [70], [71]]. Several process parameters influence the drilling performance of composite materials using ultrasonic techniques. Studies have shown that optimizing these parameters can significantly affect hole quality, including surface finish, hole diameter accuracy, and prevention of defects such as delamination and fiber pull-out [72], [73]. However, achieving the optimal combination of parameters often requires a thorough understanding of the material properties and the drilling environment. Despite its advantages, ultrasonic drilling of composite materials still faces challenges and limitations that need to be addressed for wider adoption in industrial applications. One major challenge is the selection of suitable tool materials and coatings that can withstand the high-frequency vibrations and abrasive wear encountered during drilling [[74], [75], [76], [77], [78]]. Additionally, the complex nature of composite materials, with varying fiber orientations and matrix compositions, makes it difficult to predict and control the drilling process accurately [79], [80]. Future research directions in this field include the development of advanced multi-axis ultrasonic drilling systems for complex geometries, optimization of tool designs for specific composite materials, and integration of artificial intelligence and machine learning techniques for process optimization and defect detection [81]. Furthermore, efforts to reduce process costs, improve tool life, and enhance sustainability through eco-friendly cooling/lubrication methods will be crucial for the continued advancement and adoption of ultrasonic drilling technology.

• **Abrasive Water Jet drilling (AWJ)**

AWJ drilling is a non-traditional machining process that utilizes a high-pressure jet of water mixed with abrasive particles to precisely cut through composite materials, offering a cold and burr-free drilling process ([[82], [83], [84], [85]]). AWJ drilling has emerged as a promising machining technique for composites, offering advantages such as minimal thermal damage, high precision, no tool wear, and the ability to process complex geometries without the need for secondary operations ([[86], [87], [88], [89]]). Various parameters influence the effectiveness of AWJ drilling of composite materials, including abrasive type, abrasive concentration, standoff distance, traverse speed, and water pressure ([90], [91]). Researchers have investigated the effects of these parameters on material removal rate, surface quality, and dimensional accuracy ([[92], [93], [94], [95], [96]]). Optimization of these parameters is crucial to achieving efficient and precise machining of composites ([97], [98]). Despite its advantages, AWJ drilling of composite materials faces challenges such as tapering, edge quality, and delamination, particularly in drilling thick composite stacks or laminates. To reduce drilling defects, Saini [99] developed mathematical models for surface roughness and kerf taper of abrasive jet machined nanocomposite using artificial neural network. Future research directions include the development of advanced nozzle designs, adaptive control systems, and hybrid machining approaches to address these challenges and further enhance the efficiency and reliability of AWJ drilling for composites.

• **Laser drilling**

Laser drilling has emerged as a promising technique for machining composite materials due to its ability to provide precise and controlled material removal with minimal damage to the surrounding structure ([100], [101]). The advantages of laser drilling, such as high precision, minimal thermal damage, and flexibility, are contrasted with challenges such as cost, processing time, and edge quality. Choi et al. [102] demonstrated the application of laser drilling in the aerospace industry for producing high-quality holes in CFRP composite panels used in aircraft structures. Researchers have made significant advancements in laser drilling techniques to enhance efficiency and quality. Several authors investigated the use of lasers for drilling Carbon Fiber Reinforced Polymer (CFRP) composites ([[103], [104], [105], [106], [107], [108]]). Also, several studies investigate various factors influencing the efficiency of laser drilling in composite materials ([[109], [110], [111], [112], [113], [114], [115], [116]]). The application of picosecond lasers for drilling CFRP composites was explored, showing improved hole quality and reduced heat-affected zones (HAZ) compared to traditional nanosecond lasers ([[117], [118], [119], [120]]). Tao et al. [121] investigated dual-beam laser drilling to reduce delamination in CFRP composites, achieving significant reductions in delamination and improved hole quality by controlling the distribution of laser energy.

Table 1 outlines the advantages and the challenges encountered in the literature when drilling composite materials via various drilling methods.

**Table 1:** Advantages and challenges of drilling composite materials in diverse categories.

	Advantages	Challenges
**Conventional drilling (**[34]**-** [54]**)**	• Simple • Widely available equipment	• Delamination • Fiber damage • Heat generation
**Ultrasonic drilling (**[55]**-** [81]**)**	• Reduced heat generation • Minimal fiber damage • Improved precision	• Limited to certain material thicknesses • Require specialized equipment
**AWJ drilling (**[82]**-** [99]**)**	• Cold machining process • Minimal heat-affected zone • Ability to cut through thick and hard composite materials, no tool wear	• Complexity of equipment • Maintenance • Potential for abrasive residue • Limitations in achieving very fine details
**Laser drilling (**[100]**-** [121]**)**	• High precision • Minimal physical contact with the material • Ability to create complex hole geometries	• Potential for heat-affected zones • Limited effectiveness on certain materials • Cost of laser equipment

## 3 Methodology and approach for data collection

In this section, the methodology and approach for drilling composite materials data collection involve a systematic and comprehensive review of relevant literature. The primary objective is to identify and analyze scholarly publications that contribute to the understanding of drilling techniques applied to composite materials.

In this paper, a rigorous and structured search strategy is employed in order to identify pertinent literature. Scopus academic databases are used. Indeed, a combination of keywords, including "composite materials," "drilling," and related terms, are used, ensuring a broad coverage of the research landscape. Clear criteria for including and excluding documents based on relevance are established. Also, the publications, that do not directly contribute to the understanding of drilling techniques or are not focused on composite materials, are excluded. Consequently, the following query has been used in scopus in order to identify a representative corpus of documents related to drilling of composite materials:

TITLE-ABS-KEY ((("composite materials" OR "CFRP" OR "GFRP" OR "thermosets" OR "thermoplastics")) AND (("drilling" OR "drill" OR "abrasive water jet" OR "laser" OR "ultrasonic")) AND (("damage") OR ("delamination") OR ("defects"))) AND (LIMIT-TO (SRCTYPE , "j")) AND (LIMIT-TO (DOCTYPE , "ar") OR LIMIT-TO (DOCTYPE , "re")) AND (EXCLUDE (PUBYEAR , 2024)) AND (LIMIT-TO (LANGUAGE , "English"))

This query limits the results to the English language and the year of publication up to 2023. Also, the documents are limited to journal articles and reviews. Consequently, the papers published in international conferences are excluded. The research record included “Title, Author keywords, Abstract”. The total recorded results performed on January 11, 2024 are 1010 documents, after excluding 2 duplicated documents. After checking the titles and the abstracts of all found papers, 83 articles not directly related to the searched topic were filtered and removed from the list. The study is then made on 927 papers considered for the analysis and related to drilling of composite materials. Using these selected papers, the software “VOSviewer” is utilized as tool to quantitatively assess the literature. Metrics such as number of papers and citations are taken into consideration to provide insights into the impact and evolution of research in the field of drilling of composite materials. Also, network analysis techniques are applied to identify key authors, countries, and collaborations within the research domain. The quality of the selected publications is highlighted by considering the citation rates and the reputation of the authors. The extracted data are synthesized and the findings in the context of the research objectives are interpreted. The emerging trends and the gaps in the literature as well as the areas requiring further investigation in the field of drilling of composite materials are finally identified.

## 4 Results and discussion

In this section, the results of the performance analysis conducted on the scientific papers related to drilling of composite materials are initially developed and analyzed. After that, the science mapping results of these papers are presented and discussed.

a Performance analysis

**Fig. 1:**
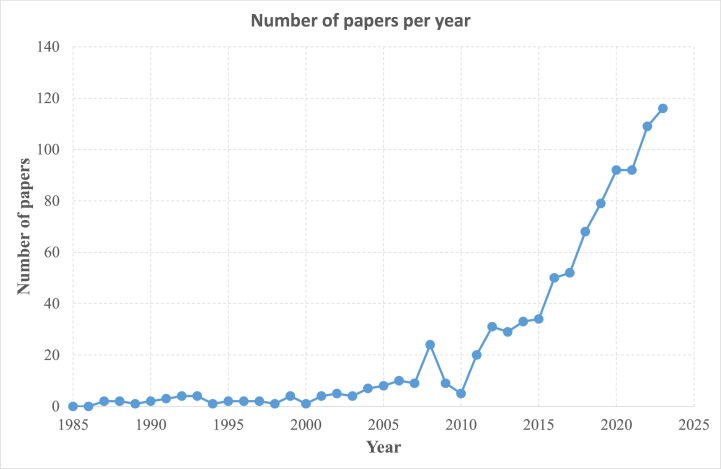
Evolution in yearly published papers on drilling of composite materials.

Over time, scientific and research interest in the drilling of composite materials has surged. Fig. 1 tracks the trajectory of published scientific papers on this topic from 1985 to 2023. Notably, the total number of papers stays below five until 2003. However, from 2004 onwards, the publication count escalates, hitting a pinnacle of 116 papers in 2023. Approximately 79 % of these publications have emerged in the last decade, underscoring the field's contemporary relevance and the need for further inquiry. A notable increase in the number of publications is observed after 2010. This surge can be attributed to several factors. First, the period after 2010 saw a significant expansion in the application of composite materials across various industries, including aerospace, automotive, and renewable energy sectors. This increased demand for composite materials necessitated more in-depth research into their machining processes, particularly drilling, which is critical for component assembly. Additionally, advancements in composite materials, such as the development of new fiber-reinforced polymers and hybrid composites, created a need for updated drilling techniques and methodologies. This led to a higher number of research activities aimed at addressing the challenges associated with drilling these advanced materials, such as delamination and tool wear. Moreover, the global push towards more sustainable and efficient manufacturing processes has driven research into optimizing drilling techniques to minimize waste and energy consumption. This trend is reflected in the growing number of publications focused on sustainable drilling practices and the development of eco-friendly technologies in the machining of composites. The recent surge is largely attributable to the growing application of composite materials across multiple sectors, such as industry, aviation, automotive, and maritime ([[122], [123], [124]]). In Fig. 1, the steady increase in published papers highlights the growing importance of drilling composite materials. Despite this increase, some areas remain underexplored. For instance, while CFRPs and GFRPs dominate research, hybrid composites and alternative drilling methods like laser or ultrasonic drilling are less represented. This indicates a potential research gap.

**Fig. 2:**
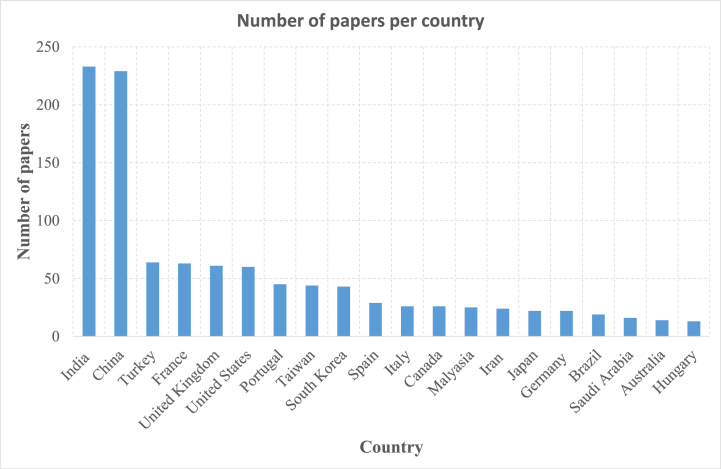
Total number of published papers per country on drilling of composite materials between 1985 and 2023.

Fig. 2 illustrates the total number of published papers per country on drilling of composite materials between 1985 and 2023. It can be noticed that the highest number of papers is found in India and China, with 233 and 229 papers, respectively. For the other countries, the total number of papers varies from 64 in Turkey to 13 in Hungary. Both European countries and the United States have significant contributions to composite materials drilling research. Moreover, the far eastern countries have also published a substantial number of papers on this topic. Although countries like India and China lead in the number of publications, some regions such as Eastern Europe and Africa have fewer contributions. Future research could benefit from increased international collaboration and focus on these underrepresented regions.

It is important to note that the prevalence of publications from a particular country can be influenced by various factors such as the number of researchers, investment in research and development, the focus of academic institutions, government policies, and more. Indeed, Both India and China have large populations, which naturally leads to a larger pool of researchers and academics. More, over the past few decades, both countries have significantly increased their investment in research.

**Fig. 3:**
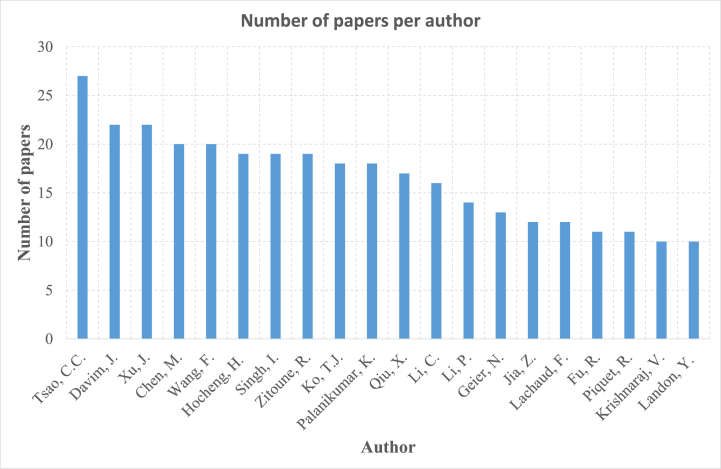
Contribution of authors to the publishing of papers on drilling of composite materials.

On the contrary, Fig. 3 illustrates the collaborative effort of authors in the publication of research papers in this specific field. By 2023, Tsao C.C. has been a leading figure in this regard, having published 27 scientific papers relating to the drilling of composite materials, which stands as the highest number among the authors. Trailing behind Tsao C.C., Davim J. and Xu J. have contributed with 22 papers each. Krishnaraj V. and Landon Y. published 10 papers each. The collaborative network shows a concentration of key authors. However, the relatively low number of publications by other researchers suggests that expanding collaboration and diversifying research teams could enhance innovation and address the existing research gaps.

**Fig. 4:**
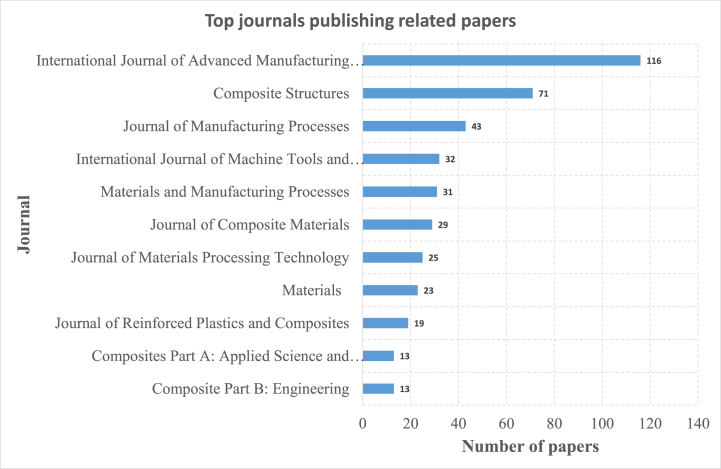
Top journals publishing on drilling of composite materials.

On the other hand, Fig. 4 illustrates the top international journals publishing on the drilling of composite materials, based on the total number of published papers on this topic. The 'International Journal of Advanced Manufacturing' takes the lead in publications with a total of 116 published papers. 'Composite Structures' follows with 71 papers published, while 'Journal of Manufacturing Processes' published 43 papers. The journals 'International Journal of Machine Tools and Manufacture', 'Materials and Manufacturing Processes', 'Journal of Composite Materials', 'Journal of Materials Processing Technology', 'Materials', 'Journal of Reinforced Plastics and Composites', 'Composites Part A: Applied Science and Manufacturing', and 'Composite Part B: Engineering' each contributed between 13 and 32 papers, as shown on Fig. 4.

**Fig. 5:**
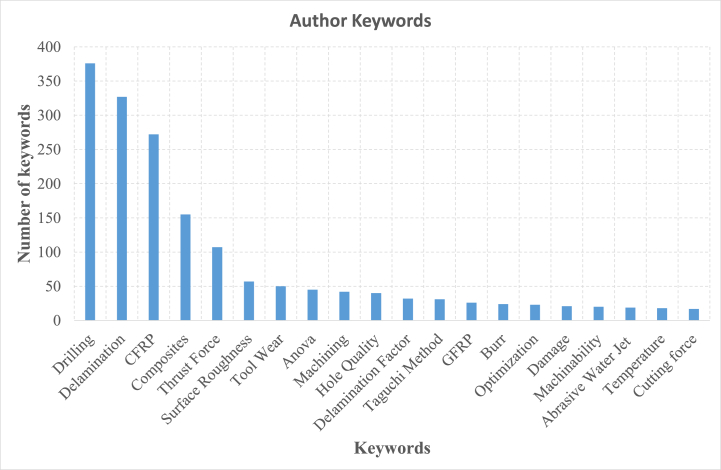
Most frequently utilized keywords by authors in published works.

As for the keywords, Fig. 5 presents the most frequently used keywords by authors in the publication related to drilling of composite materials. “Drilling” keyword is the most used one with 376 papers.

The keywords associated with defects or surface quality in drilling composite materials such as “Delamination”, “Surface Roughness”, “Hole Quality”, “Delamination Factor”, “Burr” and “Damage” are commonly utilized. The term “Delamination” alone appears in over 327 scientific works. Conversely, keywords related to materials like “CFRP”, “Composite”, and “GFRP” are cited less frequently. It is noteworthy that keywords in relation with drilling methods other than traditional cutting drilling such as “Ultrasonic Drilling”, “Abrasive Water Jet”, and “Laser Drilling” are not widely employed. It is clear on Fig. 5 that most keywords focus on conventional tools, materials and defects. There is a significant gap in understanding the wear mechanisms of advanced tools used in alternative drilling methods. More, all drilling defects in the literature are identified after drilling and research lacks comprehensive studies on real-time defect detection techniques in advanced drilling. Finally, the analysis indicates a growing interest in sustainability. However, practical implementations in drilling composite materials are suggesting another research gap.

b Science mapping

Bibliometric mapping is designed to assess academic outputs such as publication and citation information within a specific field using statistical methods [125]. The most common units of analysis in science mapping are journals, documents and authors [126]. The relationships among these units can be represented as a graph or network, where the units are depicted as nodes or circles, and the connections between them represent links. In this study, the VOSviewer software was utilized to generate bibliometric network maps illustrating relationships among keywords, documents, authors, and countries [127].

**Fig. 6:**
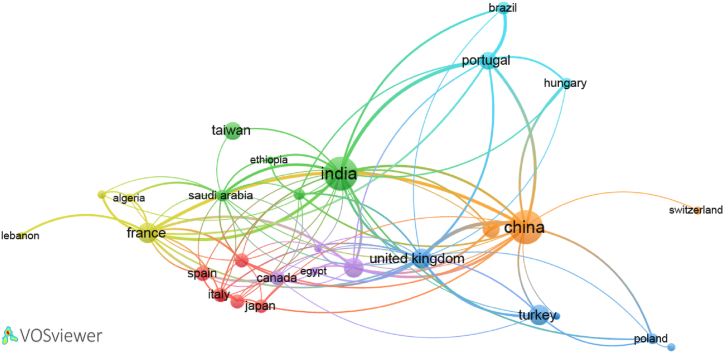
Bibliometric map based on the network of co-authorship relations among 30 countries.

Fig. 6 shows the network of international co-authorship among 30 countries, represented by 30 nodes. Each country is represented by a label and a circle, with the size of the label and circle indicating its importance. The size of each circle corresponds to the number of papers written by authors from that country. 105 existing links between circles of different countries signify co-authorship between organizations in those countries. Among these countries, and as mentioned before, India ranks first in documents production on the drilling of composite materials, with 233 articles and 20 links. China ranks second with 229 documents. Among the European countries, France ranks first with 63 documents and 17 links. Finally, these 30 countries are divided into 7 clusters, as follow.

1) Cluster 1: Iran, Italy, Japan, Malaysia, Spain and Sweden,
2) Cluster 2: Australia, Ethiopia, India, Saudi Arabia and Taiwan,
3) Cluster 3: Pakistan, Poland, Romania, Turkey and United Kingdom,
4) Cluster 4: Algeria, France, Lebanon and Tunisia,
5) Cluster 5: Canada, Egypt, United Arab Emirates and United States,
6) Cluster 6: Brazil, Hungary and Portugal,
7) Cluster 7: China, South Korea and Switzerland.

**Fig. 7:**
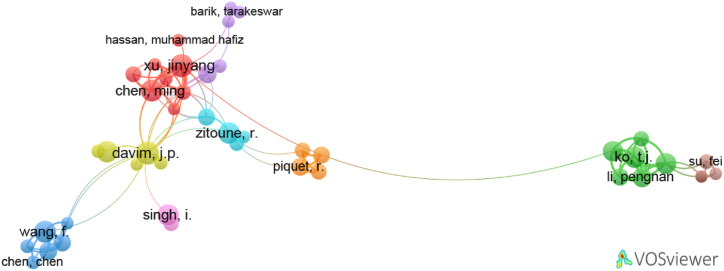
Largest network of connected authors publishing on drilling composite materials.

In the realm of publication on drilling composite materials, a total of 81 authors have been identified. Each author within the group of these authors has published a minimum of 5 papers. However, not all of them are interconnected. The largest interconnected group of authors comprises 43 individuals, as shown in Fig. 7. This group is further divided into 9 clusters, interconnected by 97 links. It is noteworthy that collaboration among authors in this field is relatively limited. The clusters of authors are presented below.

1) Cluster 1: An Quiglong, Chen Ming, El Mansori Mohamed, Hassan Muhammad Hafiz, Ji Min, Li Chao, Ren Fei, Xu Jinyang,
2) Cluster 2: Ko TJ, Li Changping, Li Pengnan, Li Shujian, Niu Qiulin, Qiu Xinyi,
3) Cluster 3: Bai Yu, Chen Chen, Fu Rao, Jia Zhenyaun, Wang Fuji, Zhang Chong,
4) Cluster 4: Abrao Alexandre Mendes, Davim JP, Latha B, Palanikumar, Reis Pedro,
5) Cluster 5: Barik Tarakeswar, Geier Norbert, Pal Kamal, Patra Karali, Pereszlai Csongor,
6) Cluster 6: Gururaja Suhasini, Krishnaraj, Kumar Dhiraj, Zitoune Redouane,
7) Cluster 7: Lachaud Frederic, Landon Yann, Piquet Robert, Rahme Pierre,
8) Cluster 8: Deng Zhaohui, Su Fei, Sun Fujian,
9) Cluster 9: Debnath Kishore and Singh Inderdeep.

**Fig. 8:**
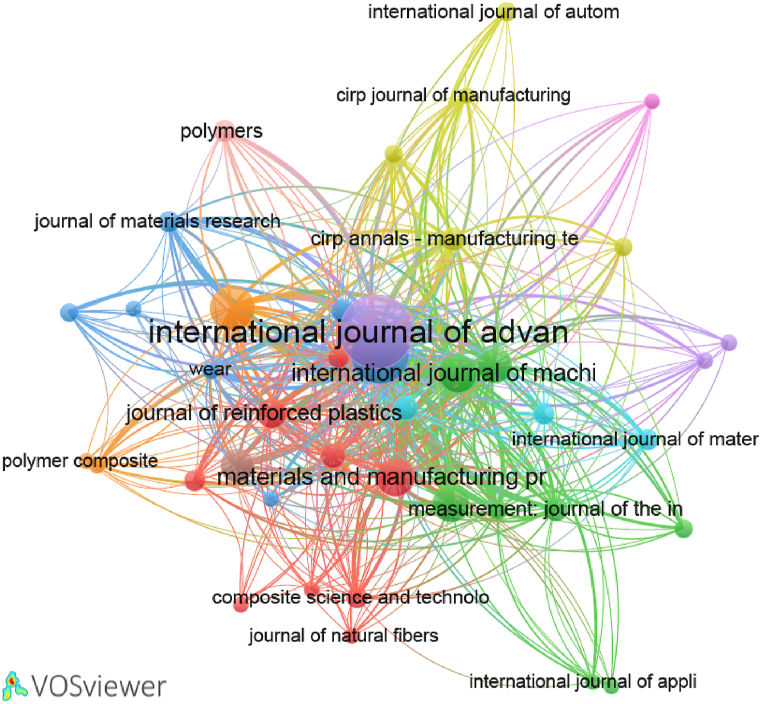
Bibliometric map based on the network of journals citation.

On the other hand, Fig. 8 illustrates the bibliometric map network of journals citation in the field of drilling composite materials. It shows again that the “International Journal of Advanced Manufacturing” is the leading publication, indicating its prominent role in disseminating research and advancements in this domain. Following closely is “Composite Structures”, showcasing its significant involvement in the field. The “Journal of Manufacturing Processes” has also made a notable impact in publications. Additionally, several other journals have contributed considerably. This distribution indicates a well-dispersed interest and research activity across multiple reputable journals, underscoring the interdisciplinary and collaborative nature of research in the drilling of composite materials. The varied contribution across these journals also highlights the diverse aspects and applications being explored within this research area. These journals are divided into 10 clusters, with 438 links of connection citations between them. Four clusters out of ten include more than five international journals.

**Fig. 9:**
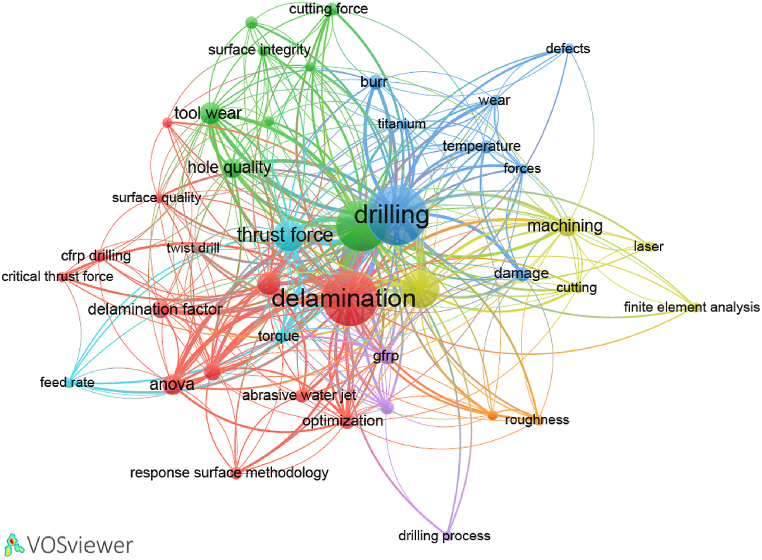
Keywords used by authors and their co-occurrence relationships.

The illustration in Fig. 9 depicts the representation of mainstream research keywords and their co-occurrence relationships. This representation contains 42 nodes representing keywords utilized by authors, organized into 8 clusters interconnected by 337 links. It highlights the prevalent terms within the realm of drilling composite materials. Node size correlates with the frequency of occurrence for each keyword. Notably, “drilling” emerges as the most frequent keyword, occurring 376 times with 38 links, followed by “delamination” with 327 occurrences and 41 links. As anticipated, keywords associated with drilling, composite materials and related defects dominate. These keywords exhibit the highest values of Total Link Strength (TLS), indicating their strong associations with other nodes ( [128], [129]). Conversely, non-conventional drilling techniques such as “ultrasonic drilling”, “abrasive water jet”, “laser”, and “orbital drilling” are underutilized. Analysis reveals eight distinct clusters of authors keywords. The first cluster encompasses 12 keywords focusing on defects, hole surface quality, and method of analysis. The second cluster comprises 8 terms emphasizing ultrasonic drilling, hole quality, and tool wear. Similarly, the third cluster, with 8 keywords, centers on drilling defects, temperature, and wear. The fourth cluster consists of 5 items relating to “composite”, “cutting”, “finite element analysis”, “laser”, and “machining”. The remaining clusters, 5 through 8, contain 3, 3, 2, and 1 keyword(s), respectively.

Consequently, the field of drilling composite materials has advanced significantly, yet several research gaps persist. This bibliometric analysis reveals a concentration on specific composite types, such as CFRP and GFRP, with less emphasis on other composites like hybrid or natural fiber composites. More, studies predominantly focus on traditional drilling techniques, leaving alternative methods like laser or ultrasonic drilling underexplored. Also, the optimization of drilling parameters is well-studied, but the understanding of tool wear mechanisms and their effects on surface integrity needs further investigation. The influence of drilling-induced damage on long-term performance and the development of real-time monitoring systems for defect detection also remain inadequately addressed. On the other hand, future research should focus on the further development of automation-based systems for real-time monitoring and control of drilling processes. Investigations into advanced signal processing techniques, such as wavelet packet analysis, for predicting tool wear and optimizing process parameters are essential. The integration of these systems with sustainable drilling practices presents an exciting avenue for research, aiming to enhance both the efficiency and environmental friendliness of composite material drilling. The application of wavelet packet analysis and machine learning algorithms has shown promise in predicting drilling defects. Integrating such methodologies can significantly improve process monitoring and reduce quality control issues. Additionally, hybrid composite-metal stack drilling, particularly involving CFRP/Ti6Al4V stacks, is increasingly common in sophisticated fabrication processes. The literature provides valuable data on how varying cutting sequences can impact drilling outcomes, emphasizing the importance of selecting appropriate strategies to minimize tool wear and enhance surface integrity. Integrating such advanced drilling techniques can lead to significant improvements in the durability and performance of drilled composite materials.

Finally, the integration of environmental sustainability considerations, such as the recyclability of drilled composites and eco-friendly lubrication techniques, is an emerging yet insufficiently explored area. This analysis underscores the need for diversified research efforts to enhance the efficiency and applicability of drilling processes in various composite materials.

## 5 Conclusion

This study provides a thorough bibliometric analysis of drilling in composite materials, using a dataset of 927 publications to map out the current research landscape. The analysis identifies key contributors, thematic trends, and collaborative networks within the field, offering a comprehensive overview of the advancements and future directions. It provides a foundational understanding of the current state of research in drilling composite materials, identifying both existing achievements and areas ripe for future exploration. While CFRP and GFRP dominate the research focus due to their widespread industrial applications, there is a clear opportunity to explore other composite types, such as hybrid and natural fiber composites, which remain underrepresented in the literature. This gap highlights the potential for diversifying future research to uncover new applications and innovations. Traditional drilling techniques are well-studied, particularly in optimizing cutting parameters, tool geometries, and material properties. However, alternative drilling methods such as laser, ultrasonic, and AWJ drilling, which offer advantages in reducing delamination and improving surface quality, require further exploration. Future studies should assess the industrial applicability and benefits of these alternative methods. The study also underscores the importance of understanding tool wear mechanisms and their impact on surface integrity. While optimization of drilling parameters is crucial, there is a pressing need for advanced automation, real-time monitoring systems, and innovative cutting strategies, especially in hybrid composite-metal stack drilling, to enhance surface integrity and reduce tool wear. Furthermore, addressing drilling-induced damage remains a key challenge. Delamination, fiber pull-out, thermal damage, and other defects significantly affect the long-term performance of composite materials. Real-time defect detection systems are essential for improving quality control and ensuring the reliability of composite structures. Sustainability in drilling practices, including the recyclability of composites and eco-friendly lubrication techniques, is an emerging yet underexplored area. As industries push for more sustainable practices, research in this direction will be vital for future innovations. The bibliometric analysis reveals a concentration of research output in regions like India and China, indicating a need for broader global collaboration and knowledge exchange to leverage diverse expertise.

## Data availability statement

The data that support the findings of this bibliometric review are derived from publicly available datasets. The primary sources of data include Scopus Database. Access to Scopus requires a subscription or institutional access. The datasets generated during and/or analyzed during the current study are available from the corresponding author on reasonable request.

## Ethics statement

This bibliometric review was conducted with the highest ethical standards in mind. The following ethical considerations were adhered to throughout the research process.

• All data used in this review were obtained from publicly accessible databases and sources. No proprietary or confidential information was used.
• The authors declare no conflict of interest in the preparation and publication of this paper.
• Proper attribution has been given to all original sources of data, and all intellectual property rights have been respected. The review cites all sources accurately and comprehensively.
• Efforts were made to ensure the accuracy and integrity of the data analyzed. Any limitations or potential biases in the data sources or methods have been transparently reported.
• This research did not receive any specific grant from funding agencies in the public, commercial, or not-for-profit sectors.
• The study complies with the ethical guidelines and standards set forth by the Lebanese American University (LAU), as well as relevant ethical standards in bibliometric research.

## CRediT authorship contribution statement

**Pierre Rahme:** Writing – review & editing, Writing – original draft, Visualization, Validation, Supervision, Software, Methodology, Investigation, Formal analysis.

## Declaration of competing interest

The authors declare that they have no known competing financial interests or personal relationships that could have appeared to influence the work reported in this paper.
